# Epidemiology of Trauma Patients from the Mosul Offensive, 2016–2017: Results from a Dedicated Trauma Center in Erbil, Iraqi Kurdistan

**DOI:** 10.1007/s00268-018-4817-1

**Published:** 2018-10-24

**Authors:** Maximilian P. Nerlander, Rawand Musheer Haweizy, Moayad Abdullah Wahab, Andreas Älgå, Johan von Schreeb

**Affiliations:** 1 Centre for Research on Health Care in Disasters, Department of Public Health Sciences, Karolinska Institutet, 171 76 Stockholm, Sweden; 2 College of Medicine, Hawler Medical University, Erbil, Iraq; 3 Kurdistan Board of Medical Specialties, Erbil, Iraq

## Abstract

**Introduction:**

Most epidemiological studies from conflicts are restricted to either combatants or civilians. It is largely unknown how the epidemiology differs between the two groups. In 2016, an Iraqi-led coalition began retaking Mosul from the terrorist group Islamic State of Iraq and Syria. One key institution that received trauma patients from Mosul was Emergency Management Center (EMC) in Erbil, 90 km away. The aim of this study was to describe the epidemiology, morbidity, and mortality of civilians and combatants admitted during the ongoing conflict.

**Method:**

This retrospective cohort study utilized routinely collected data on patients with conflict-related injuries who were admitted to EMC between October 16, 2016, and July 10, 2017. Data processing and analysis was carried out using JMP 13. Categorical variables were compared using Fisher’s exact test.

**Results:**

The analysis included 1725 patients, out of which 46% were civilian. Ordnance accounted for most injuries (68%), followed by firearms (18%) and improvised explosive devices (IEDs) (14%). The proportion of IED-related injuries among combatants were almost three times that of civilians. The proportions of abdominal injuries, need for surgery, laparotomies, and amputations were significantly higher among civilians than among combatants. The mortality rate was 0.5%.

**Discussion:**

The fact that civilians had greater surgical needs than combatants may be explained by several factors including a lack of ballistic protection. The extremely low mortality rate indicates significant gaps in prehospital care and transport. Our results may provide useful information to guide medical preparedness and response during future conflicts.

**Clinicaltrials.gov ID:**

NCT03358758.

## Introduction

Between October 16, 2016, and July 10, 2017, an internationally supported coalition involving the Iraqi Security Forces (ISF), Kurdish Peshmerga, and Shia militias reclaimed the city of Mosul from the terrorist organization Islamic State of Iraq and Syria (ISIS). Estimates of total fatalities during the offensive vary. A report by the UN Assistance Mission for Iraq and the Office of the UN High Commissioner for Human Rights estimates that 2521 civilians were killed, while aggregate data compiled by the Associated Press estimate that there were between 9000 and 11,000 civilian fatalities [1, 2].

The total number of patients who were in need of surgery remains unknown, but a lack of advanced trauma care close to the conflict was a significant problem, especially during the first months of fighting. During this time, injured patients had to be transported to Erbil for surgical trauma care, 90 km away. It took several months until a functional trauma care system, provided by national and international agencies, was in place. The system included prehospital trauma stabilization points (TSP), emergency surgical trauma care, ambulance referrals, and referral surgical trauma care. A report by the Johns Hopkins Bloomberg School of Public Health (BSPH) estimates that between 1500 and 1800 lives were saved thanks to these efforts [2].

Several studies conducted in the Eastern Mediterranean region have investigated the epidemiology of conflict-related trauma. A recent study from the Syrian conflict using mortality data from the Syrian Violation Documentation Center found that the mechanism of fatal injury to civilians varied with the stage of the conflict [3]. US military studies indicated that explosions were a leading cause of battlefield trauma in combatants [4–6]. However, to date, there are few published articles that directly compare the epidemiology of conflict-related trauma in civilians with that of combatants. When conducted, these tend to focus on a particular injury mechanism or on a specific anatomical injury site [7–10].

Injury mechanism, severity of injury, and in-hospital mortality all have implications for clinical management and resource allocation. Therefore, an expanded understanding of how the epidemiology of conflict-related trauma differs between civilians and combatants may be of value to those involved in providing surgical trauma care to any patients during modern conflicts.

This study aims to describe and compare the epidemiology, morbidity, and mortality of civilian and combatant casualties admitted to a specialized trauma care facility during the Mosul offensive.

## Methods

Emergency Management Center (EMC), located approximately 90 km east of Mosul in Erbil, Iraqi Kurdistan, is a dedicated trauma center and one of the key medical institutions that received trauma patients from the Mosul offensive. This facility is a 108-bed referral hospital with diagnostic capabilities including computed tomography scanners, which provides general, orthopedic, and vascular surgery. EMC systematically collected data on admitted patients. A paper-based file followed the patient throughout the hospital stay. Upon discharge or death, the file was brought to a central archive. Data were manually extracted from existing files and registries to produce a line list in Microsoft Excel which was subsequently cleaned and analyzed using JMP 13. Variables included demographics, date of admission, date of discharge, injury mechanism, anatomical injury site, status as civilian or combatant, operative interventions, and length of intensive care unit (ICU) stay. Results were stratified according to standard age categories and combatant status. Categorical variables were compared using Fisher’s exact test, where a *p* value of <0.05 was considered significant.

The inclusion criteria for this study were any trauma patient admitted to EMC between October 16, 2016, and July 10, 2017, with an injury that was sustained in Mosul and attributable to the conflict. Two categories of patient *identity* were used. A *combatant* was defined as a person belonging to either the ISF or the Peshmerga, the main military force of Iraqi Kurdistan. A c*ivilian* was defined as a person reportedly not belonging to a warring faction and not actively participating in combat. Identity was assigned by the emergency room nurse based on the presence or absence of a uniform, the presence of a combat casualty card, and self-report. Three *injury mechanisms* were identified. A *firearm* was any weapon firing nonexplosive kinetic projectiles. *Ordnance* included any conventional explosives, including grenades, mortars, and rocket-propelled grenades. *Improvised explosive devices (IEDs)* were a heterogeneous family of explosives consisting of weaponized explosive materials. Together, ordnance and IEDs are referred to as *explosives*. Injury mechanism was assigned based on injuries identified by the physician and information from the ambulance services, self-report, or witnesses to the incident. In terms of outcome, a fatality was defined as a patient dying after having arrived at the hospital. Injury severity was estimated using proxy markers, including need for surgery, type of surgery, and duration of ICU admission.

Ethical permission was obtained from the Research Ethics Committee, Kurdistan Regional Government (Id: 632017).

## Results

Data were collected on a total of 1832 consecutive patients meeting the inclusion criteria. Out of these, 107 patients were excluded due to their identity being neither combatant nor civilian. Examples include police officers and private security personnel. Thus, a total of 1725 patients were included in the final analysis.

The majority (87%) of patients were male. Most patients were identified as combatant (54%), followed by civilian (46%). Most patients had been injured by ordnance (68%) followed by firearms (18%) and IEDs (14%). The overall in-hospital mortality rate was 0.5% (Table 1).

**Table 1:** Demographics, morbidity, mortality, and surgeries of patients presenting to EMC between October 16, 2016, and July 10, 2017, by any route of admission, with injuries sustained in Mosul, where the injury is attributable to the conflict and of a violent nature (*n* = 1725)

	*n* (%)
*Sex*	
Male	1507 (87)
Female	218 (13)
*Identity*	
Combatant	932 (54)
Civilian	793 (46)
*Injury mechanism*	
Ordnance	1176 (68)
Firearm	306 (18)
IED	243 (14)
*Anatomical injury site*	
Extremities	1091 (58)
Trunk	418 (22)
Head/neck^a^	360 (19)
*In-hospital deaths*	8 (0.5)

Surgeries	*n*
Total	1031
Debridement	315
Laparotomy	71
Amputation	28
Thoracotomy	1

^a^Includes facial and eye injuries. Multiple injuries to different structures counted separately

**Fig. 1:**
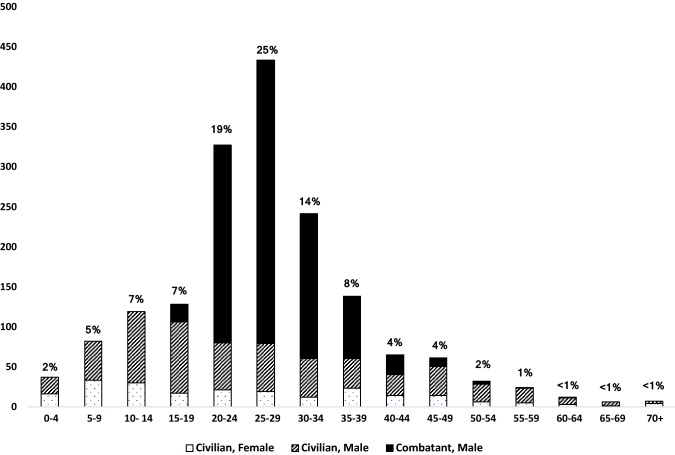
Age distribution and combatant status (*n* = 1725). A total of 13 individuals whose age was unknown were excluded from the age analysis but included in the analysis pertaining to sex and combatant status

In all standard age categories except 70+, the majority of patients were male, even with combatants excluded. There were no female combatants represented in this dataset. The three most common age categories were 20–24, 25–29, and 30–34, and males in these categories constituted more than half of total patients (Fig. 1).

The proportion of firearm-related injuries were similar between civilians and combatants. Civilians were more likely to have been injured by ordnance, while combatants were almost three times more likely to have been injured by IEDs. Civilians were more than twice as likely to have abdominal injuries as compared to combatants. The proportion of patients undergoing surgery among civilians was more than five times that of combatants. Civilians were more than six times as likely to undergo amputation and eight times more likely to undergo a laparotomy. Among patients who required ICU care, 21% of civilians required 10 days or more in ICU, while only one combatant was in the ICU this long (Table 2).

**Table 2:** Injury mechanism, location of injury, surgery, and ICU care by status, % of combatants and civilians (*n* = 1725)

	Combatant, *n* (%)	Civilian, *n* (%)	*p* value
*Injury mechanism*			
Firearm	178 (19)	128 (16)	0.11
Ordnance	570 (61)	606 (76)	<0.05
IED	184 (20)	59 (7)	<0.05
*Anatomical injury site* ^a^			
Head/neck^b^	193 (21)	156 (20)	0.63
Upper limb	237 (25)	167 (21)	<0.05
Lower limb	328 (35)	359 (45)	<0.05
Abdomen	64 (7)	127 (16)	<0.05
Thorax	48 (5)	57 (7)	0.09
Back	65 (7)	57 (7)	0.93
*Required surgery*	95 (10)	418 (53)	<0.05
*Type of surgery* ^c^			
Laparotomy	11 (1)	60 (8)	<0.05
Amputation	4 (0.4)	24 (3)	<0.05
*Length of ICU care* ^d^			
≥10 days	1 (3)	14 (21)	<0.05

^a^Denominator includes 59 patients where anatomical injury site was unknown

^b^Includes facial and eye injuries. For comparative analysis, multiple injuries to different structures are counted as one injury

^c^Among patients requiring surgery

^d^Among patients requiring ICU care (*n* = 105)

## Discussion

Males aged 20–39 years constituted more than half of the patients, and the majority of these patients were combatants. Similar observations have been made elsewhere in Iraq. Analysis of data from the Iraqi National Injury Surveillance System revealed that men in the very same age span constituted a majority of all fatal injuries from firearms [11]. The results must also be understood in the context of what is known about the wider trauma care response to the Mosul operation. The BSPH report estimates that out of 19,784 patients presenting to a total of 10 facilities during the Mosul operation, a slight majority (55%) of patients were female [2]. Since the BSPH report includes facilities close to the battlefield, this may indicate that more males than females were referred to higher-level institutions, as males predominated in our study population.

Explosives were the leading injury mechanism among civilians and combatants alike. Studies conducted in conflicts elsewhere in the Middle East have made similar observations. A recent retrospective study of US military casualties in Iraq found that more than three quarters of casualties were due to explosives [4]. Similarly, a report by the U.S. Department of Veteran Affairs determined that 74% of casualties in the U.S. Joint Theatre Trauma Registry were due to explosives [6]. Data from Syria describing the cause of fatalities among civilians indicate that in the early stages of the conflict, firearm-related injuries were responsible for the majority of civilian fatalities, while a greater proportion of civilians were killed by explosives at later stages [3].

Civilians were more than twice as likely to sustain abdominal injuries as compared to combatants and significantly more likely to undergo laparotomies and amputations. This may have been attributable to the ballistic protection worn by combatants, which usually protects the torso through anterior and posterior steel or ceramic armor plate inserts, as well as access to armored vehicles which provide additional protection. The effect of the discrepancy in access to protection was twofold. First, civilians were at greater risk of injuries to the abdomen. Second, when injuries did occur, they were more likely to be severe due to the absence of protection, increasing the likelihood of a laparotomy being required.

Civilians were more than five times more likely to require any surgery overall and were also more likely to require lengthy ICU care. This may be reflective of ISF evacuation policies, where wounded ISF service members were evacuated to EMC, stabilized, and transported to Baghdad within 24 h. Thus, nonurgent surgeries in this patient group were not conducted at EMC, and length of ICU care was limited.

The in-hospital mortality rate was lower than what has generally been observed in other settings. By comparison, studies from urban hospitals in India, a setting with comparable healthcare resources to Iraqi Kurdistan but with different types of injuries, reveal significantly higher in-hospital trauma fatality rates. A retrospective study of trauma patients in Mumbai revealed a fatality rate of 8.1% during the first hours following admission [12]. Further, a study of 11,671 trauma admissions in five urban Indian hospitals revealed an overall 30-day mortality of 21.6%, while a separate study of 11,202 trauma admissions found an in-hospital mortality of 21.4% [1, 13]. In the UK, a mortality study of 66,734 trauma patients involved in mass casualty events revealed an overall ER mortality of 6.5–7.6% [14]. Our results therefore suggest that the most critically injured patients died before reaching the hospital due to long transport times and significant shortcomings in prehospital care. During the Mosul operation, patients were transported in Iraqi ambulances, driven to the Kurdish–Iraqi border where a security check was conducted, then transferred to Kurdish ambulances, and driven to Erbil. This coupled with limited healthcare provision close to the battlefield, especially during the early stages of the campaign, implies that many patients may have died prior to reaching EMC. This is supported by studies investigating the epidemiology of trauma patients in Kunduz, Afghanistan, a conflict setting with significant shortcomings in prehospital care, which found in-hospital mortality rates between 0.1 and 12.6% [15, 16]. The very low incidence of thoracic injuries and thoracotomies in this study is also an indication of a high prehospital mortality, since traumatic intrathoracic events such as tension pneumothoraces and hemothoraces can rapidly be fatal unless care is initiated early [17].

### Limitations

This study was subject to a number of limitations. First, it was an analysis of data from a single center and therefore not representative of all trauma patients during the Mosul operation. Unpublished data from West Emergency Hospital, another hospital in Erbil, suggest that this facility cared for additional 2608 patients. Second, since the conflict involved irregular forces with no consistent uniform use, distinguishing combatants from civilians may have been challenging, particularly in unconscious patients who were unable to self-report. Third, due to the database in this analysis having been compiled from handwritten records, collection was subject to transcription error. Fourth, the data were subject to survival bias since, due to long transportation times and gaps in prehospital care, only patients well enough to reach care were included. As a result, critical injuries were underrepresented, which limits the generalizability for settings closer to the battlefield. Lastly, patient demographics may have been unreliable, since some patients come from socioeconomic settings where the date of birth may not have been recorded. However, due to the random nature of these uncertainties, any effect on the analysis is likely to have been limited.

## Conclusion

This study was based on data collected from a dedicated trauma center providing a leading role in meeting healthcare needs during the offensive against ISIS in Mosul and as such represents the first investigation of its kind from this particular conflict. We have demonstrated that in the context of the Mosul operation, civilians and combatants that were referred to EMC had suffered different types of injuries and that civilians may have been more likely to have suffered severe injuries. The low in-hospital mortality indicates that there may be gaps in prehospital care and transportation. Additional studies conducted closer to the battlefield are needed in order to better understand what factors determine the epidemiology of conflict-related trauma patients in the early phase following injury and to what extent the availability of early resuscitation of trauma patients affects mortality.
